# EphB2 mediates social isolation-induced memory forgetting

**DOI:** 10.1038/s41398-020-01051-6

**Published:** 2020-11-09

**Authors:** Xin-Rong Wu, Yu Zhang, Xian-Dong Liu, Wu-Bo Han, Nan-Jie Xu, Suya Sun

**Affiliations:** 1 Department of Neurology and Institute of Neurology, Ruijin Hospital, Shanghai Jiao Tong University School of Medicine, 200025 Shanghai, China; 2 Collaborative Innovation Center for Brain Science, Department of Anatomy and Physiology, Shanghai Jiao Tong University School of Medicine, 200025 Shanghai, China; 3 Key Laboratory of Cell Differentiation and Apoptosis of the Chinese Ministry of Education, Shanghai Jiao Tong University School of Medicine, 200025 Shanghai, China; 4 Shanghai Key Laboratory of Reproductive Medicine, Shanghai Jiao Tong University School of Medicine, 200025 Shanghai, China

**Keywords:** Molecular neuroscience, Hippocampus

## Abstract

Social isolation in adolescence leads to lasting deficits, including emotional and cognitive dysregulation. It remains unclear, however, how social isolation affects certain processes of memory and what molecular mechanisms are involved. In this study, we found that social isolation during the post-weaning period resulted in forgetting of the long-term fear memory, which was attributable to the downregulation of synaptic function in the hippocampal CA1 region mediated by EphB2, a receptor tyrosine kinase which involves in the glutamate receptor multiprotein complex. Viral-mediated EphB2 knockdown in CA1 mimicked the memory defects in group-housed mice, whereas restoration of EphB2 by either viral overexpression or resocialization reversed the memory decline in isolated mice. Taken together, our finding indicates that social isolation gives rise to memory forgetting by disrupting EphB2-mediated synaptic plasticity, which may provide a potential target for preventing memory loss caused by social isolation or loneliness.

## Introduction

Accumulating evidence over the past century, from social psychology to behavioral neuroscience, indicates the importance of social bonds for the survival of social species^1,2^. The social connection ensures safety and security, supports the survival of offspring, and reduces the need for energy expenditure^3^. Conversely, the absence of social interaction such as social isolation (SI), which is an aversive state in humans, is detrimental to physical and mental well-being^4^. SI can produce numerous behavioral, morphological, and functional abnormalities in the central nervous system^5,6^. In particular, post-weaning SI has been regarded as a risk factor for mental health problems during adolescence^4,6,7^.

SI contributes to poorer cognitive performance, faster cognitive decline, poorer executive functioning, increased negativity, and depressive cognition^8^. Increasing evidence shows that post-weaning isolation can impair spatial memory and social memory^9–11^. However, it remains unclear whether the memory impairments are due to the disruption of encoding, recalling information, or forgetting of events. Among the cognitive nuclei, the hippocampus serves as a functional core to integrate external environmental stimuli and intrinsic circuit activity for memory formation, which does it by receiving inputs from dentate granule cells and sending outputs through CA1 cells to process information^12^. CA1 neuron functions relying on subregional synaptic transmission/remodeling and also participates in the wiring of inter-nucleus circuits from the hippocampus to the cortex/amygdala^13–15^, which is crucial for fear memory. The dendritic spine, a specialized structure in neurons, plays a key role in synaptic remodeling for cellular function. Structural abnormalities of dendritic spines may contribute to synaptic dysfunction and have been implicated in memory impairment^16–19^. The synaptic function depends on the number and location of postsynaptic receptors, especially the glutamate receptor multiprotein complex, as well as their expression and signal properties^20–22^.

As a key component of the glutamate receptor multiprotein complex, EphB receptors promote synapse formation and maturation by organizing functional presynaptic specializations^23^, guiding axons^24^, inducing spine morphogenesis, and recruiting neurotransmitter receptors^25^. As critical molecules in the developmental phase^26^, EphB receptors also participate in a variety of functional events in cognition and memory^27–29^.

In this study, we found that post-weaning SI caused a rapidly forgetting of long-term memory. Furthermore, we identified the critical role of hippocampal EphB2 in SI-caused memory forgetting.

## Methods

### Mice

*Thy1-GFP-M*^30^ transgenic mice and *EphB2*^*−/−*^^31^ knock-out mice genotyping methods have been described previously. The mice (both males and females) were backcrossed to the 129 strain. All experiments involving mice were carried out in accordance with the US National Institutes of Health Guide for the Care and Use of Animals under an Institutional Animal Care and Use Committee approved protocol in an Association for Assessment and Accreditation of Laboratory Animal Care–an approved facility at the Shanghai Jiao Tong University School of Medicine.

### Social isolation

**Fig. 1:**
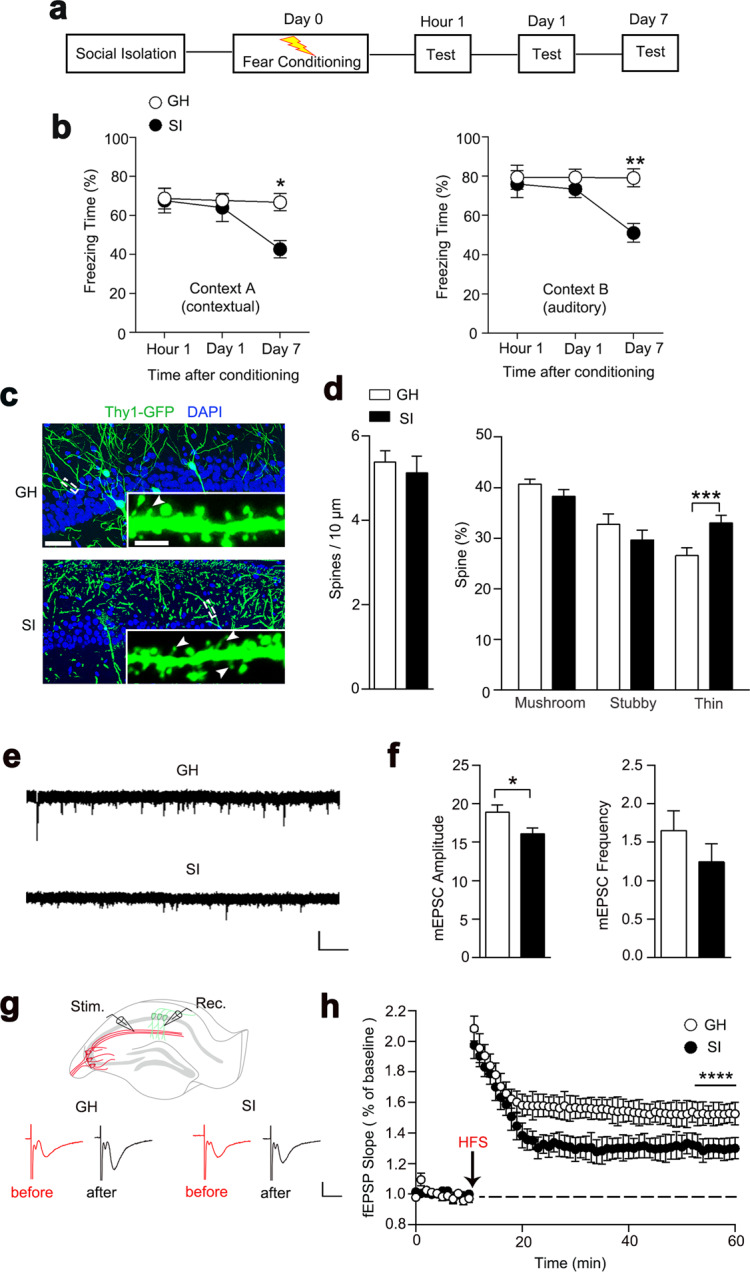
Four-week isolated mice showed the forgetting of long-term fear memory. **a** Experimental paradigm for the memory process in fear conditioning. **b** Long-term memory was impaired in SI (4 W) mice compared with GH mice. GH, *n* = 11 mice; SI, *n* = 7 mice, two-way *ANOVA*, context A, effect of time, *F*_(2, 57)_ = 3.522, *P* < 0.05; effect of group, *F*_(1, 57)_ = 4.767, *P* < 0.05; group × time, *F*_(2, 57)_ = 2.743, *P* > 0.05. Tukey multiple-comparison test: hour 1: *P* > 0.05; day 1: *P* > 0.05; day 7: *P* < 0.05. Context B, effect of time, *F*_(2, 57)_ = 3.432, *P* < 0.05; effect of group, *F*_(1, 49)_ = 8.377, *P* < 0.01; group × time, *F*_(2, 57)_ = 3.284, *P* < 0.05. Tukey multiple-comparison test: hour 1: *P* > 0.05; day 1: *P* > 0.05; day 7: *P* < 0.01. **c** Representative immunostaining of spines (green) and DAPI (blue) from GH and SI mice. Arrows indicate thin-shaped spines. Scale bar: 50 μm, 2.5 μm (inset). **d** Quantification of the densities for total spines, thin spines, mushroom spines, and stubby spines. GH, *n* = 20 neurons from five mice; SI, *n* = 18 neurons from four mice; unpaired Student’s *t* test, spines density, *t*_(36)_ = 0.5472, *P* > 0.05; mushroom spines, *t*_(36)_ = 1.075, *P* > 0.05; stubby spines, *t*_(36)_ = 1.128, *P* > 0.05; thin spines, *t* = 3.136, *P* < 0.01. **e**, **f** The mEPSC was recorded in CA1 neurons from GH and SI mice (left). Right panel shows the average mEPSC amplitude and frequency. Calibration: 15 pA, 1 s. GH, *n* = 16 neurons from four mice; SI, *n* = 15 neurons from four mice; unpaired Student’s *t* test, mEPSC amplitude, *t*_(29)_ = 2.293, *P* < 0.05; mEPSC frequency, *t*_(29)_ = 1.165, *P* > 0.05. **g** Schematic diagram in the top panel shows fEPSP recordings of the CA3-CA1 pathway. The stimulating electrode was placed in the Schaffer of CA3, and a recording pipette was placed in the stratum radiatum of area CA1. Stim stimulating electrode, Rec recording pipette. Representative fEPSP trace at baseline and last 10 min in LTP recordings. Calibration: 0.5 mV, 10 ms. **h** Quantification of the average fEPSP slope during the last 10 min of the LTP recording from GH and SI mice. GH, *n* = 9 slices from three mice; SI, *n* = 8 slices from three mice; two-way ANOVA; effect of group, *F*_(1, 165)_ = 51.72, *P* < 0.0001; effect of time, *F*_(10, 165)_ = 0.02342, *P* > 0.05; group × time, *F*_(10, 165)_ = 0.01884, *P* > 0.05. All data are presented as mean ± SEM. *P < 0.05; **P < 0.01; ***P < 0.001; ****P < 0.0001.

For the whole program, mice were randomly divided into two groups: group housing (GH) with three or four mice per cage and social isolation (SI) with only one mouse per cage. In the beginning of the experiment, we isolated the mice from postnatal week 4 (PW4) for different time durations: 2, 4, and 12 weeks (Fig. 1 and Supplementary Fig. 1). After the following behavioral experiments, a 4-week social isolation model was chosen in all behavioral trials.

### Fear conditioning and extinction

All tests were conducted according to the previous study^32^. All mice underwent handling and had 1 h to habituate the behavioral room before any tests began. The conditioning chambers (17 × 17 × 25 cm) equipped with stainless-steel shocking grids were connected to a precision feedback current-regulated shocker (Ugo Basile, Italy). The chamber walls were covered with black-and-white checkered wallpaper (context A), and the chambers were cleaned with 75% ethanol. On the day before the fear-conditioned training, mice were placed in context A to explore freely for 20 min and then returned to their home cages. On the training day, mice were also placed in context A to explore freely for 3 min first, then a pure tone (CS) was played for 28 s, followed by an electric foot shock (US, 0.75 mA, 2-s duration) through the floor grid. One minute later, paired CS–US repeated. Conditioned mice were returned to their home cages 30 s after the end of the last tone, and the floor and walls of the cage were cleaned with 75% ethanol for each mouse. One hour after conditioning, mice were placed in context A to test the freezing time (contextual fear memory). Thirty minutes later, animals received CS-alone presentations in a test chamber, which had gray non-shocking plexiglass floor and dark gray wallpaper and was washed with 4% acetic acid solution between the tests for individual mice (context B). In detail, the mice explored freely for 3 min, and then the tone was played continually for 1 min in context B. We analyzed the freezing behavior during tone presentations. The freezing time in context B refers to auditory fear memory. We tested the contextual and auditory fear memory on days 1 and 7 after conditioning. In extinction experiments, we used 3-consecutive-day extinction in this study. Animals were presented with unreinforced exposures to the conditioning context. Mice behaviors were recorded by digital video cameras mounted above the conditioning chamber. FreezeFrame and FreezeView software (Ugo Basile, Italy) were used for recording and analyzing the freezing behavior, respectively.

### Electrophysiology

Brain coronal slices were prepared from GH mice and 4-week SI mice. Brains were dissected quickly and chilled in ice-cold artificial cerebrospinal fluid (ACSF) containing (in mM): 125 NaCl, 2.5 KCl, 2 CaCl_2_, 1 MgCl_2_, 25 NaHCO_3_, 1.25 NaH_2_PO_4_, and 12.5 glucose. Coronal brain slices (300-μm thick) were prepared with a vibratome and recovered in ACSF bubbled with 95% O_2_ and 5% CO_2_ at 31 °C for 1 h and then maintained at room temperature (22–25 °C). Miniature excitatory postsynaptic potential current (mEPSC) was recorded at −70 mV in the presence of 100 μM picrotoxin and 1 μM tetrodotoxin. For PPR recording, slices were stimulated using a bipolar concentric electrode (FHC) that was placed in the CA1 and connected with a stimulator (AMPI) to evoke EPSCs (recorded in 100 μM picrotoxin ACSF solution) in neighboring pyramidal neurons. PPRs were calculated as a ratio of EPSC2 to EPSC1, separated by interstimulus intervals of 25, 50, 100, 200, and 400 ms. For long-term potentiation (LTP) recording, extracellular field excitatory postsynaptic potentials (fEPSPs) in the Schaffer collateral pathway were synaptically evoked and recorded in the CA1 region. LTP was induced using high-frequency stimulation (HFS) consisting of 1-se 100 Hz trains, each delivered at 70–80% of the intensity that evoked spiked fEPSPs. Data were analyzed in pCLAMP 10.6 (Molecular Devices), and recordings were made from an average of three cells per slice and two to three slices per mouse.

### Stereotaxic surgery

Mice were subjected to the operative procedure using an aseptic technique. The virus of AAV-EphB2 or lenti-EphB2 shRNA (Lenti-sh-EphB2, sequence ACGGACAAGCTACAACACT)^33^ were stereotaxically injected into CA1 (1.7 mm posterior and 1.25 mm left or right to the bregma, and 1.55 mm deep into the skull surface). After injection, the hand-held needle was kept in place for an additional 5 min to avoid the backflow of the virus containing solution. Following injection, the mice were collected on a 37 °C warm plate for recovery. Mice were allowed to recover for 25–30 days before starting the experiments. At the end of the experiment, mice used for behavioral tests were perfused and checked for the injection sites; off-target virus injection mice were excluded when we performed the statistical analysis.

### Immunohistochemistry

Immunofluorescence was performed as described in our previous study^34^, coronal brain slices were blocked with permeable buffer (0.3% Triton X-100 in PBS) containing 10% donkey serum for 1 h at room temperature and were incubated with primary antibodies in permeable buffer containing 2% donkey serum overnight at 4 °C. The slices were then washed three times with PBS-T (0.1% Tween-20 in PBS) for 10 min each and incubated with Alexa Fluor secondary antibodies (1:200; Molecular Probes) in the PBS buffer for 2 h at room temperature. Slices were washed in PBS-T three times and photographed using a confocal microscope (Leica TCS SP8). Fluorescence microscopic images obtained were imported into Image J (NIH) for analysis, and all the parameters used were kept consistent during capture. For primary antibodies, we used goat anti-EphB2 (1:200, R&D, P54763). To quantify the shape of spine, a procedure was adapted from our previous study^33^. The shape of neuronal spines in slices was classified by NeuronStudio software package and an algorithm from Rodriguez^35^ with the following cutoff values: aspect ratio for thin spines (AR_thin _(crit)_) = 2.5, head-to-neck ratio (HNR _(crit)_) = 1.3, and head diameter (HD _(crit)_) = 0.3 μm. The type of these spines was determined based on the following criteria: (a) spines with HNR greater than HNR _(crit)_ were considered to have a neck and could be either thin or mushroom types; (b) spines with HD greater than HD _(crit)_ were classified as mushroom, otherwise thin; (c) spines lacking significant necks and less than AR_thin _(crit)_ were considered stubby, otherwise thin. Protrusions with length 0.2–3.0 μm and maximum width 3 μm were counted. Spine density was calculated by dividing the total spine number by the dendritic branch length. The fluorescence intensity of EphB2 was quantified by Image J. All the pictures were processed with the same threshold.

### Western blotting

Western blotting was performed as described in our previous study^36^. Briefly, hippocampus from GH mice and 4-week SI mice were dissected, homogenized, and solubilized at 4 °C for 1 h in lysis buffer (1% CHAPS, 137 mM NaCl, 2.7 mM KCl, 4.3 mM Na_2_HPO_4_, 1.4 mM KH_2_PO_4_, 5 mM EDTA, 5 mM EGTA, 1 mM PMSF, 50 mM NaF, 1 mM Na_3_VO_4_, and protease inhibitors, pH 7.2). Bound proteins were separated by SDS PAGE, transferred to nitrocellulose membranes, and immunoblotted with the indicated antibodies: goat anti-EphB1 (1:500, Santa Cruz Biotechnology, sc-68317), goat anti-EphB2 (1:1000, R&D, P54763), mouse anti-synaptophysin (1:1000, Abcam, ab8049), rabbit anti-PSD95 (1:1000; Cell Signaling Technology, 3450), mouse anti-β-actin (1:3000; Thermo Fisher Scientific, MA5-15739), rabbit anti-GluN1 (1:1000, BD, 556308), mouse anti-GluA1 (1:1000, Santa Cruz Biotechnology, sc-13152), GluA2 (1:1000, Santa Cruz Biotechnology, sc-7611), GluA6 (1:1000, Santa Cruz Biotechnology, sc-7618), rabbit anti-GluN2A (1:1000, Millipore, ab1555P), and mouse anti-GluN2B (1:1000, BD, 610417).

### Statistical analysis

The results are presented as mean ± SEM. Statistical differences were determined by Student’s *t* test for two-group comparisons or analysis of variance (ANOVA) followed by the Tukey test for multiple comparisons among more than two groups. A *P* value of < 0.05 was considered statistically significant.

## Results

### Social isolation induces forgetting of long-term fear memory

To determine how SI impairs the cognitive process, we analyzed the memory performance of GH and SI mice, which were isolated for 2/4/12 weeks from postnatal week 4 (PW4), using the Pavlovian fear conditioning test. The mice received tests 1 h after conditioning, which was considered as short-term memory, and 1 day and 7 days after conditioning, which was considered as long-term memory. We found that 2-week isolated mice showed normal memory in terms of both short-term memory and long-term memory (Supplementary Fig. 1a, b). In the 4-week isolated mice, memory deficits were seen 7 days after conditioning, whereas the 12-week isolated mice showed memory defects earlier, with auditory fear deficits observed even 1 day after conditioning (Fig. 1 a, b and Supplementary Fig. 1c). These results indicate that long-term memory deficits are associated with the duration of SI. We used a 4-week isolation model in the remaining experiments. Notably, we did not see either long- or short-term memory decline after repeated exposure in the control group or find a significant difference between GH and SI mice after an extinction procedure (Supplementary Fig. 1d), suggesting that memory deficits in SI mice were not likely due to the extinction. No obvious difference was seen in Y-maze spontaneous alternation, a short-term memory test (Supplementary Fig. 2a). Furthermore, we did not observe any difference in fear response between male and female mice (Supplementary Fig. 2b, c). Taken together, these results provide evidence that SI leads to a phenotype of accelerated forgetting of long-term fear memory rather than of initially acquired memory or short-term memory.

Considering the critical roles of hippocampal synaptic morphogenesis in cognition^12,16,17^, we analyzed the spinogenesis in hippocampal neurons and observed the increased number of thin-shaped spines in CA1 area of isolated mice, with no difference in total density (Fig. 1c, d). To examine the synaptic function, we measured the mEPSC with whole-cell patch-clamp electrophysiology in brain slices and observed a decreased amplitude rather than frequency of mEPSC in these neurons from SI mice (Fig. 1e, f). No significant change was observed in the presynaptic neurotransmitter release, as demonstrated by the paired-pulse ratio (Supplementary Fig. 2d, e). We further detected the LTP in the Schaffer collateral-CA1 pathway, a critical cellular mechanism of learning and memory, and we observed a decreased LTP level in the SI group compared with the GH mice, indicating that long-term isolation impairs hippocampal synaptic plasticity (Fig. 1g, h).

### EphB2 mediates the social isolation-induced memory forgetting

**Fig. 2:**
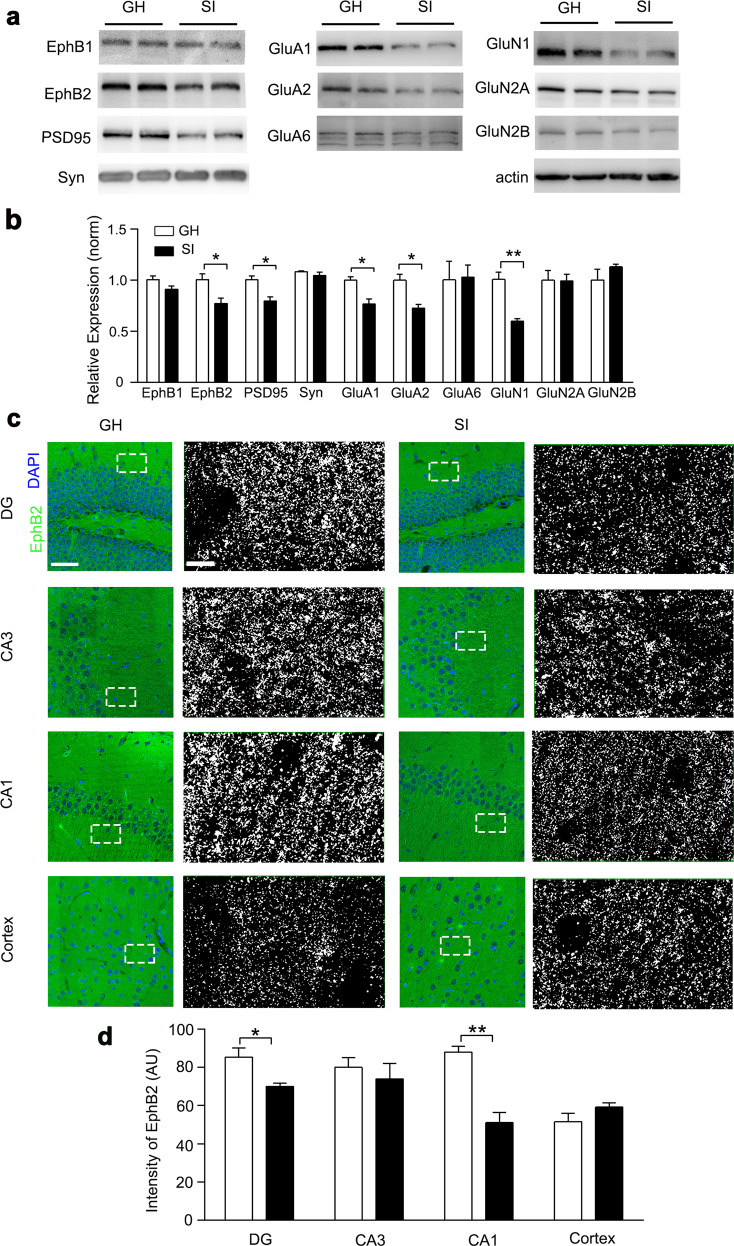
Hippocampal EphB2 was downregulated in isolated mice. **a** The expression levels of EphB1, EphB2, PSD95, syn (synaptophysin), GluA1, GluA2, GluA6, GluN1, GluN2A, and GluN2B proteins in the hippocampus of GH and SI mice. **b** Quantification of EphB1, EphB2, PSD95, synaptophysin, GluA1, GluA2, GluA6, GluN1, GluN2A, and GluN2B proteins in the hippocampus. *n* = 3 mice for each group, paired Student’s *t* test; EphB1, *t*_(2)_ = 1.960, *P* > 0.05; EphB2, *t*_(2)_ = 4.299, *P* < 0.05; PSD95, *t*_(2)_ = 2.840, *P* < 0.05; synaptophysin, *t*_(2)_ = 0.9553, *P* > 0.05; GluA1, *t*_(2)_ = 3.378, *P* < 0.05; GluA2, *t*_(2)_ = 4.063, *P* < 0.05; GluA6, *t*_(2)_ = 0.1099, *P* > 0.05; GluN1, *t*_(2)_ = 3.243, *P* < 0.05; GluN2A, *t*_(2)_ = 0.0597, *P* > 0.05; and GluN2B, *t*_(2)_ = 1.167, *P* > 0.05. **c** Representative immunostaining of EphB2 (green) and DAPI (blue) in the hippocampus and cortex of GH and SI mice. Right panel shows the details (the pictures were processed with the same threshold by Image J). Scale bar: 50 μm (left), 7.5 μm (right). **d** Quantification of EphB2 fluorescence intensity in the hippocampus and cortex. *n* = 3 mice for each group, paired Student’s *t* test, DG, *t*_(2)_ = 2.972, *P* < 0.05; CA3, *t*_(2)_ = 0.6402, *P* > 0.05; CA1, *t*_(2)_ = 6.018, *P* < 0.01; cortex, *t*_(2)_ = 1.508, *P* > 0.05. All data are presented as mean ± SEM. *P < 0.05; **P < 0.01.

The hippocampal glutamate receptor complex in the postsynaptic compartment has been implicated in cognition and synaptic function^37–39^. The complex includes glutamate receptors (AMPA receptors and NMDA receptors) and EphB receptors, which modulate excitatory synaptic transmission through protein clustering at postsynaptic membranes^23,29,40^. To figure out the molecular mechanism of isolation-induced memory defects, we tested the expression of these key proteins in hippocampal tissues extracted from GH and SI mice. We observed decreased levels of GluN1, GluA1, and GluA2, three glutamate receptor subtypes (Fig. 2a, b and Supplementary Fig. 3), and in particular, the EphB2 receptor, a key protein involved in the multiprotein complex that plays an important role in initial social recognition memory^29^. Immunofluorescence staining further showed that EphB2 in CA1 was significantly decreased in the SI group, with no apparent change in the cortex and CA3 region (Fig. 2c, d and Supplementary Fig. 4a, b).

**Fig. 3:**
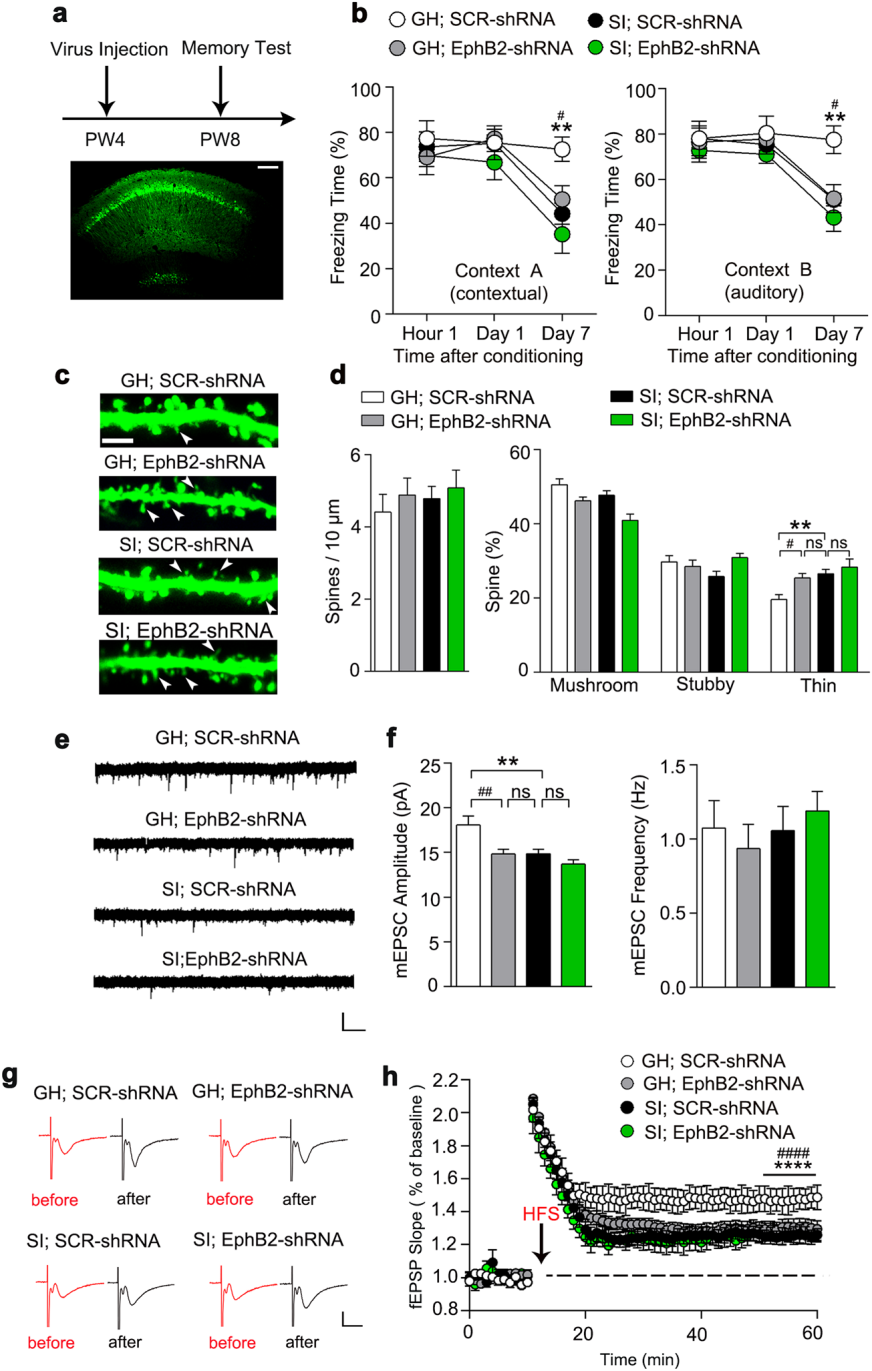
Knockdown of EphB2 mimics the cognitive deficits in GH mice. **a** Top panel shows the experimental paradigm; the bottom panel shows a representative confocal image of virus injection in CA1. Scale bar: 150 μm. **b** GH mice with EphB2-shRNA showed a decreased freezing time on day 7 after training comparable to the level of control mice in context A (left panel), and similar results were obtained in context B (right panel). GH with control virus, *n* = 8 mice; GH with EphB2-shRNA, *n* = 7 mice; SI with control virus, *n* = 8 mice, SI with EphB2-shRNA, *n* = 8 mice; two-way ANOVA; for more detailed statistical results, see Supplementary Table 1. **c** Representative immunostaining of spines (green) in CA1. Arrows indicate thin-shaped spines. Scale bar: 2.5 μm. **d** The number of thin-shaped spines was increased in GH mice after infusion of EphB2-shRNA. GH with control virus, *n* = 19 neurons from five mice; GH with EphB2-shRNA, *n* = 19 neurons from four mice; SI with control virus, *n* = 20 neurons from five mice; SI with EphB2-shRNA, *n* = 17 neurons from four mice; two-way ANOVA; for more detailed statistical results, see Supplementary Table 2. **e** The mEPSC was recorded in CA1 neurons from GH mice with SCR-shRNA, SI mice with SCR-shRNA, GH mice with EphB2-shRNA, and SI mice with EphB2-shRNA. Calibration: 15 pA, 1 s. **f** Quantification of the average mEPSC amplitude and frequency of these groups. GH with control virus, *n* = 16 neurons from four mice; GH with EphB2-shRNA, *n* = 16 neurons from four mice; SI with control virus, *n* = 16 neurons from four mice; SI with EphB2-shRNA, *n* = 15 neurons from four mice; two-way ANOVA; for more detailed statistical results, see Supplementary Table 3. **g** Representative fEPSP trace at baseline and last 10 min in LTP recordings from GH with SCR-shRNA, GH with EphB2-shRNA, SI with SCR-shRNA, and SI with EphB2-shRNA. Calibration: 0.5 mV, 10 ms. **h** Quantification of the average fEPSP slope during the last 10 min of the LTP recording. GH with SCR-shRNA, *n* = 9 slices from three mice; GH with EphB2-shRNA, *n* = 8 slices from three mice; SI with SCR-shRNA, *n* = 8 slices from three mice; SI with EphB2-shRNA, *n* = 8 slices from three mice; two-way ANOVA; for more detailed statistical results, see Supplementary Table 4. All data are presented as mean ± SEM. * means the difference between GH mice with SCR-shRNA and SI mice with SCR-shRNA, # means the difference between GH mice with SCR-shRNA and GH mice with EphB2-shRNA. *P < 0.05; **P < 0.01; ****P < 0.0001; ^#^*P* < 0.05; ^##^*P* < 0.01; ^####^*P* < 0.0001.

As EphB2 participates in memory regulation^39,41,42^, we hypothesized that the downregulation of EphB2 could mimic the behavioral abnormalities in SI mice. We thus generated a lentiviral vector expressing both green fluorescent protein (GFP) and EphB2 shRNA (Lenti-sh-EphB2) (referred to as “EphB2-shRNA”)^14^ to knock down EphB2 (Supplementary Figs. 4c, d and 5a), which allowed us to test the role of EphB2 in forgetting of long-term memory. We bilaterally injected EphB2-shRNA into the CA1 region at PW4 and observed that GH mice infused with EphB2-shRNA showed less freezing time than control mice 7 days after conditioning, which was comparable to the level of SI mice (Fig. 3a, b). Morphologically, a considerable number of thin-shaped spines in GH mice with the infusion of EphB2-shRNA was observed, which was similar to SI mice (Fig. 3c, d). Moreover, we found that knockdown of EphB2 decreased mEPSC amplitude and suppressed LTP in CA1 region of GH mice, which mimicked the synaptic dysfunction observed in SI mice (Fig. 3e–h). These results suggest that EphB2 is necessary for SI-induced memory deficits.

### Functional reversion of memory forgetting by EphB2 restoration

**Fig. 4:**
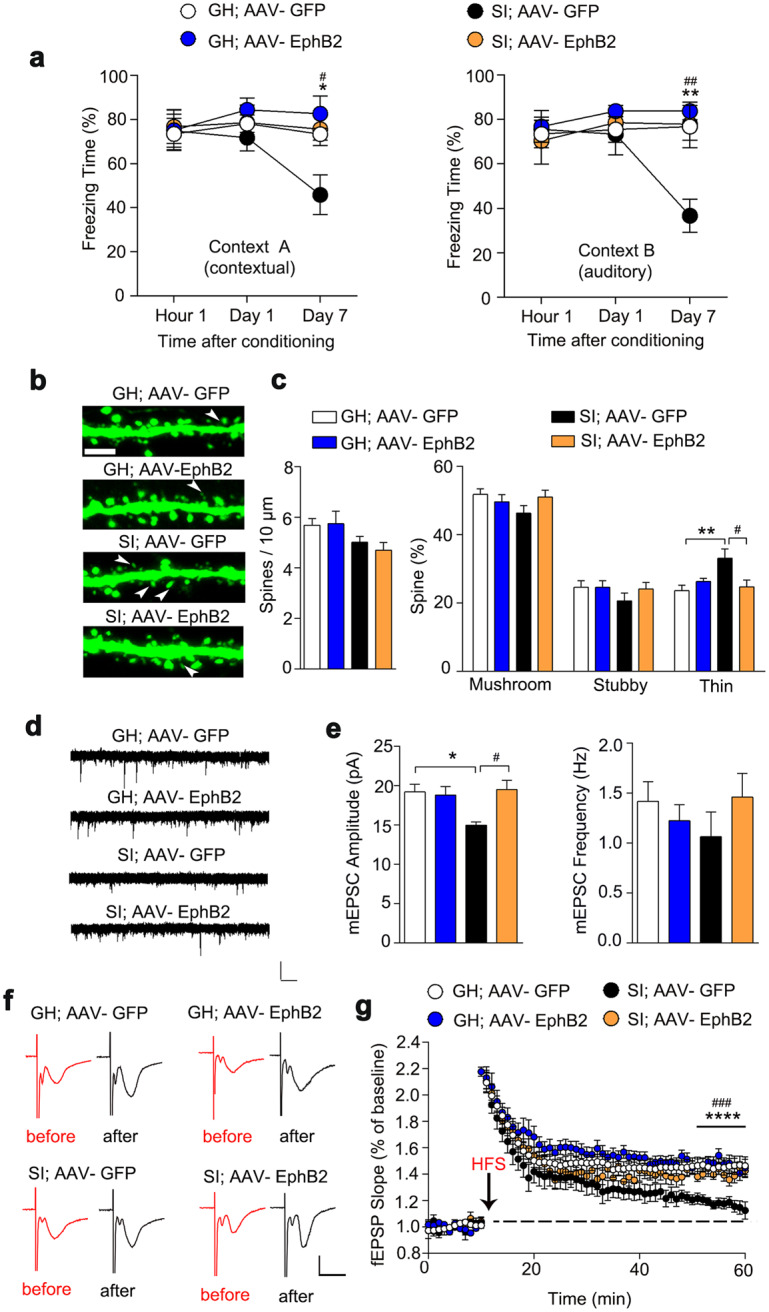
Overexpression of EphB2 rescues the cognitive deficits in isolated mice. **a** Isolated mice showed an increasing freezing time on day 7 after training with the infusion of AAV-EphB2 comparable to the level of control mice in context A (left panel), and similar results were obtained in context B (right panel). GH with AAV-GFP, *n* = 8 mice; GH with AAV-EphB2, *n* = 10 mice; SI with AAV-GFP, *n* = 8 mice, SI with AAV-EphB2, *n* = 7 mice; two-way ANOVA; for more detailed statistical results, see Supplementary Table 5. **b** Representative immunostaining of spines (green) in CA1. Arrows indicate thin-shaped spines. Scale bar: 2.5 μm. **c** The number of thin-shaped spines was decreased in SI mice after infusion of AAV-EphB2. GH with AAV-GFP, *n* = 19 neurons from five mice; GH with AAV-EphB2, *n* = 16 neurons from four mice; SI with AAV-GFP, *n* = 16 neurons from four mice; SI with AAV-EphB2, *n* = 16 neurons from four mice; two-way ANOVA; for more detailed statistical results, see Supplementary Table 6. **d** The mEPSC were recorded in CA1 neurons from GH mice with AAV-GFP, SI mice with AAV-GFP, GH mice with AAV-EphB2, and SI mice with AAV-EphB2. Calibration: 15 pA, 1 s. **e** The average mEPSC amplitude and frequency. GH with AAV-GFP, *n* = 17 neurons from four mice; GH with AAV-EphB2, *n* = 18 neurons from four mice; SI with AAV-GFP, *n* = 17 neurons from four mice; SI with AAV-EphB2, *n* = 18 neurons from four mice; two-way ANOVA; for more detailed statistical results, see Supplementary Table 7. **f** Representative fEPSP trace at baseline and last 10 min in LTP recordings from GH with AAV-GFP, GH with AAV-EphB2, SI with AAV-GFP, SI with AAV-EphB2. Calibration: 0.5 mV, 10 ms. **g** Quantification of the average fEPSP slope during the last 10 min of the LTP recording. GH with AAV-GFP, *n* = 9 slices from three mice; GH with AAV-EphB2, *n* = 8 slices from three mice; SI with AAV-GFP, *n* = 9 slices from three mice; SI with AAV-EphB2, *n* = 8 slices from three mice; two-way ANOVA; for more detailed statistical results, see Supplementary Table 8. All data are presented as mean ± SEM. * means the difference between GH mice with AAV-GFP and SI mice with AAV-GFP, # means the difference between SI mice with AAV-GFP and SI mice with AAV-EphB2. *P < 0.05; **P < 0.01; ****P < 0.0001; ^#^*P* < 0.05; ^##^*P* < 0.01; ^####^*P* < 0.01.

To further determine the sufficiency of EphB2 as a mediator of synaptic and cognitive function, we tested whether overexpression of exogenous EphB2 in CA1 could rescue the behavioral defects, morphological deficits, and impaired LTP in SI mice. Intra-CA1 administration of AAV-EphB2 resulted in a profound increase in expression levels of EphB2 (Supplementary Figs. 4e, f and 5b), which also successfully rescued long-term memory in SI mice (Fig. 4a). Furthermore, after overexpressing the exogenous EphB2, mEPSC and LTP were fully recovered (Fig. 4d–g), accompanied by a restoration of the subtype of spines in CA1 (Fig. 4b, c). These results provided additional supportive evidence for the role of EphB2 in isolation-induced forgetting of long-term memory.

**Fig. 5:**
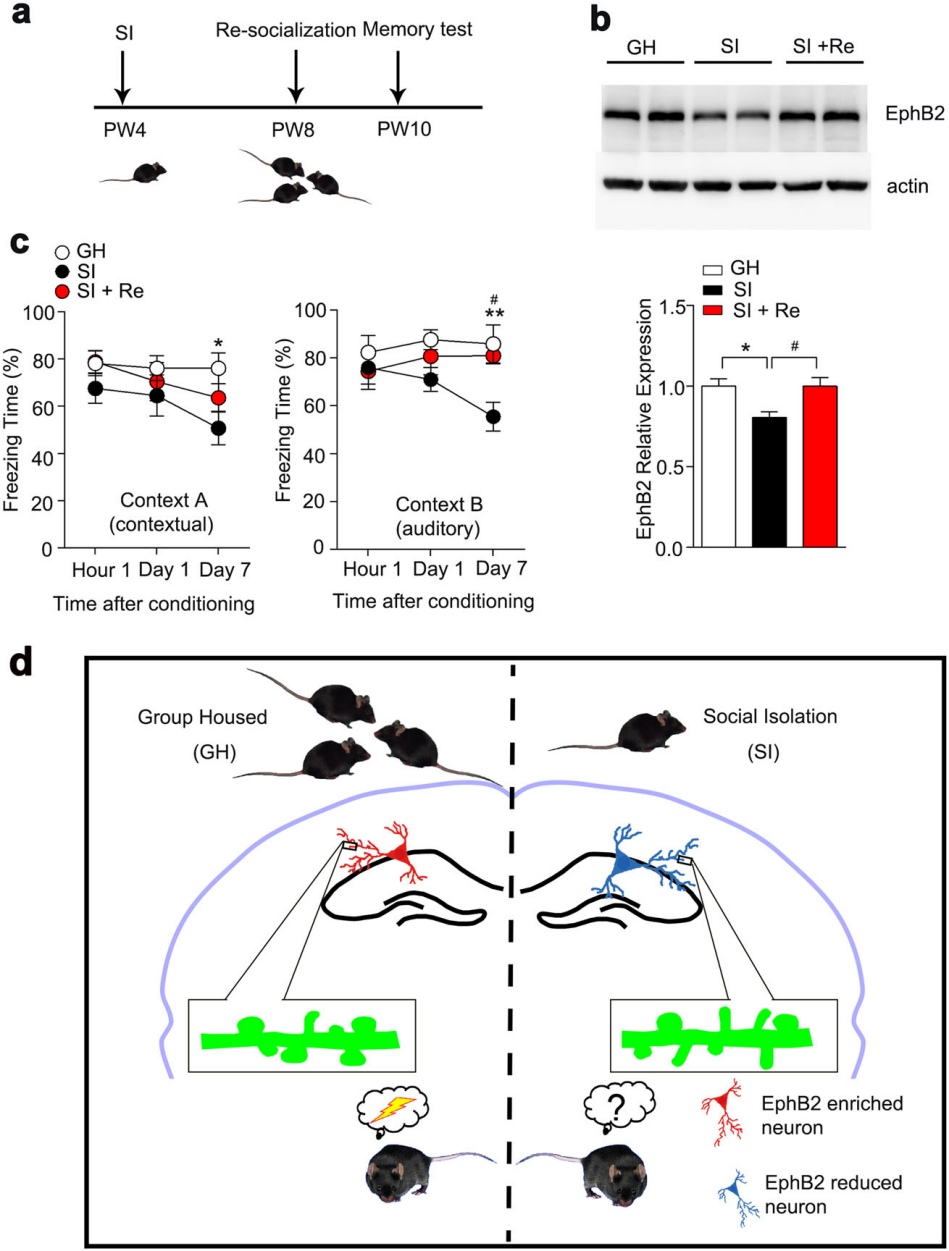
Resocialization reversed the memory impairments in isolated mice. **a** Experimental paradigm of resocialization. **b** The expression of EphB2 in the hippocampus of GH mice, SI mice, and mice with SI followed by resocialization was detected by western blotting (top panel). The bottom panel shows the quantification of EphB2 protein in the hippocampus. *n* = 3 mice for each group, one-way ANOVA, *F*_(2,9)_ = 6.186, *P* < 0.05. **c** Isolation followed by resocialization mice showed an increase freezing time on day 7 after training comparable to that of GH mice in context A (left panel), and similar results were obtained in context B (right panel). GH, *n* = 9 mice, SI, *n* = 11 mice, reGH, *n* = 7 mice, one-way factorial ANOVA, test of hour 1 in context A, *F*_(2,24)_ = 1.272, *P* > 0.05; test of day 1 in context A, *F*_(2,24)_ = 0.5017, *P* > 0.05; test of day 7 in context A, *F*_(2,24)_ = 3.794, *P* < 0.05; test of hour 1 in context B, *F*_(2,24)_ = 0.3233, *P* > 0.05; test of day 1 in context B, *F*_(2,24)_ = 2.612, *P* > 0.05; test of day 7 in context B, *F*_(2,24)_ = 7.066, *P* < 0.01. **d** Proposed model for EphB2 function in SI-induced forgetting of long-term memory. All data are presented as mean ± SEM. * means the difference between GH mice and SI mice, # means the difference between SI mice and SI mice with resocialization. *P < 0.05; **P < 0.01; ^#^*P* < 0.05.

Environment enrichment or resocialization was previously demonstrated to be beneficial for memory maintenance^43–45^ and could rescue the cognitive dysfunction induced by isolation^9,46^. We subjected the 4-week isolated mice to resocialization for 2 weeks and found that the expression of EphB2 in the hippocampus was restored in the re-socialized mice, and the freezing time in these mice also increased compared with the isolated group. These results suggest that a normal social environment can retain long-term memory through EphB2 (Fig. 5b, c). Taken together, our finding reveals the EphB2 is required and sufficient to sustain long-term memory in a social environment, which provides a molecular and synaptic mechanism for isolation-induced memory forgetting.

## Discussion

In this study, we revealed that post-weaning isolation resulted in forgetting of long-term fear memory through disruption of the synaptic function in the hippocampal CA1 region. We further demonstrated that EphB2 played an essential role in memory retention by promoting spinogenesis and synaptic plasticity (Fig. 5d). Our study is distinct from previous studies that have focused on adulthood^9^, and we provide a molecular mechanism that could control the synaptic function for the SI-induced memory forgetting during adolescence. The scientific study of memory set in with Ebbinghaus’ forgetting curve illustrating the progressive deterioration of long-term memory as time goes by^47^. Since then, much has been learned about possible causes for this gradual memory loss, but the neurobiological processes underpinning the memory forgetting remain poorly understood. SI is associated with a higher risk of cognitive deterioration and neuronal disorders^9,48,49^. Our present results suggested that SI caused the forgetting of long-term fear memory rather than deficits in short-term memory or learning ability, which was different from previous studies^10,50^. The results are in agreement with the synaptic remodeling process, in which long-term memory parallels synaptic strengthening from days to weeks, rather than short-term memory, which lasts from minutes to hours^51–53^.

We demonstrated that EphB2 played an important role in SI-induced memory forgetting, which strengthens the evidence that EphB2 is involved in enhancing synaptic transmission, and the gene expression is believed to underlie memory formation^39,41,54,55^. EphB2 interacts with NMDA receptors and regulates excitatory synapse formation. Previous studies have shown that EphB2 interacts directly with GluN1 through its extracellular region, which regulates synaptic function^56,57^. On the one hand, EphB2 enhances the phosphorylation and localization of GluN2B-containing NMDA receptor at synapses^37,58^. Considering the decreased protein level of GluN1 in the hippocampus of isolation-reared mice (Fig. 2a), we assume that the EphB2–NMDA protein complex may serve as an essential functional core in SI-induced memory deficits. On the other hand, EphB2 also regulates the trafficking of GluA2 depending on its PDZ-binding motif: The PDZ-binding motif of EphB2 binds to the PDZ domain-containing scaffold protein glutamate receptor-interacting protein 1 (GRIP1), which binds to GluA2 and regulates the localization of GluA2-containing AMPA receptors^23,40,59^. AMPA receptor-dependent endocytosis has been proposed to be an active process underlying the intrinsic forgetting of LTP and the forgetting of relevant memories^60^. Furthermore, trans-synaptic ephrin-B–EphB2 interactions and forward signaling facilitate neural activation and structural plasticity in learning-associated neurons involved in the generation of fear memory^61^. Therefore, EphB2 regulates the forgetting of long-term memory induced by SI may pass through different synaptic molecular pathways.

Together with the accumulating evidences, EphB2 mediates the excitatory synaptogenesis during development and coordinates synaptic plasticity by controlling the localization and function of glutamate receptors^27,59^. Previous studies also show that EphB2 signal is related to anxiety disorders^62^, autism^63,64^, and cognitive dysfunction^41,65^. Our study expands the understanding of the function of EphB2 in the social environment and memory, which may bring about new insights for the treatment of these cognition-associated brain disorders.

## Supplementary information

Supplementary Fig
