# Supplementary material for: EphB2 mediates social isolation-induced memory forgetting
Source: Transl Psychiatry. 2020 Nov 9;10:389. doi: 10.1038/s41398-020-01051-6 (PMC7653962; doi:10.1038/s41398-020-01051-6)
Supplement: Supplementary file 1 — Supplementary Fig [file 41398_2020_1051_MOESM1_ESM.pdf]

EphB2 mediates social isolation induced memory forgetting

Xin-Rong Wu[1, 5], Yu Zhang[2, 5], Xian-Dong Liu[1,2], Wu-Bo Han[1], Nan-Jie Xu[2,3,4*], Suya Sun[1*]

Supplementary Figures and Tabels

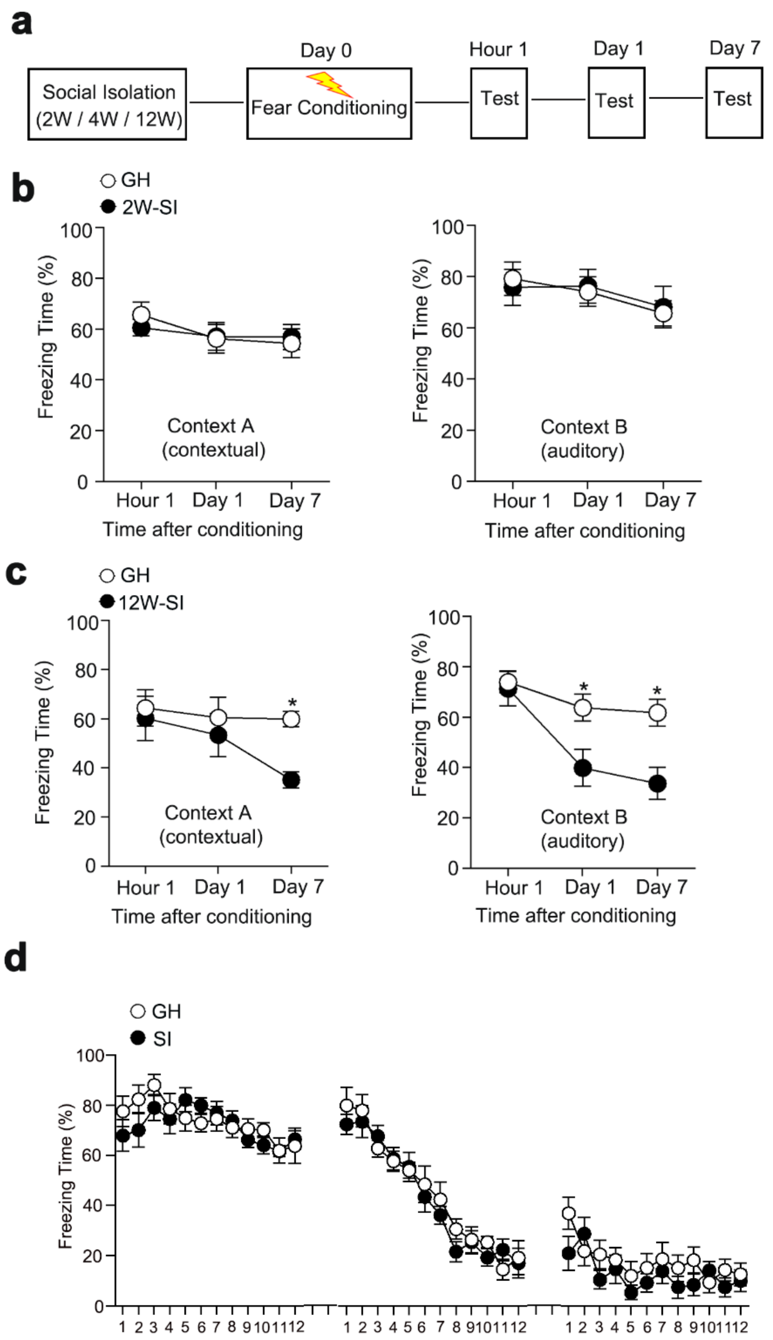

Supplementary Fig. 1 SI induces the forgetting of long-term fear memory.

a Experimental paradigm of memory process in fear conditioning. **b** No difference was seen in short-term memory or long-term memory between GH mice and SI (2W) mice

in fear conditioning. GH, $n = 12$ mice; SI, $n = 12$ mice, two-way *ANOVA*, context A, effect of time, $F_{(2, 66)} = 1.277$, $p > 0.05$; effect of group, $F_{(1, 66)} = 0.01826$, $p > 0.05$; group × time, $F_{(2, 66)} = 0.3063$, $p > 0.05$; context B, effect of time, $F_{(2, 66)} = 1.428$, $p > 0.05$; effect of group, $F_{(1, 66)} = 0.01826$, $p > 0.05$; group × time, $F_{(2, 66)} = 0.1231$, $p > 0.05$, Tukey post hoc analysis was performed. **c** Long-term memory was impaired in SI (12W) mice compared with GH mice. GH, $n = 11$ mice; SI, $n = 7$ mice, two-way *ANOVA*, context A, effect of time, $F_{(2, 49)} = 2.134$, $p > 0.05$; effect of group, $F_{(1, 49)} = 4.163$, $p < 0.05$; group × time, $F_{(2, 49)} = 1.184$, $p > 0.05$, Tukey post hoc analysis was performed; context B, effect of time, $F_{(2, 49)} = 10.2$, $p < 0.001$; effect of group, $F_{(1, 49)} = 14.19$, $p < 0.001$; group × time, $F_{(2, 49)} = 2.668$, $p > 0.05$; Tukey post hoc analysis was performed. **d** No difference was seen in fear extinction between GH and SI mice. GH = 7 mice, SI = 7 mice, two-way *ANOVA*, effect of time, $F_{(35, 432)} = 59.46$, $p < 0.0001$; effect of group, $F_{(1, 432)} = 6.980$, $p < 0.01$; group × time, $F_{(35, 432)} = 0.7259$, $p > 0.05$, Tukey post hoc analysis was performed. All data are presented as mean ± SEM. $^*p < 0.05$.

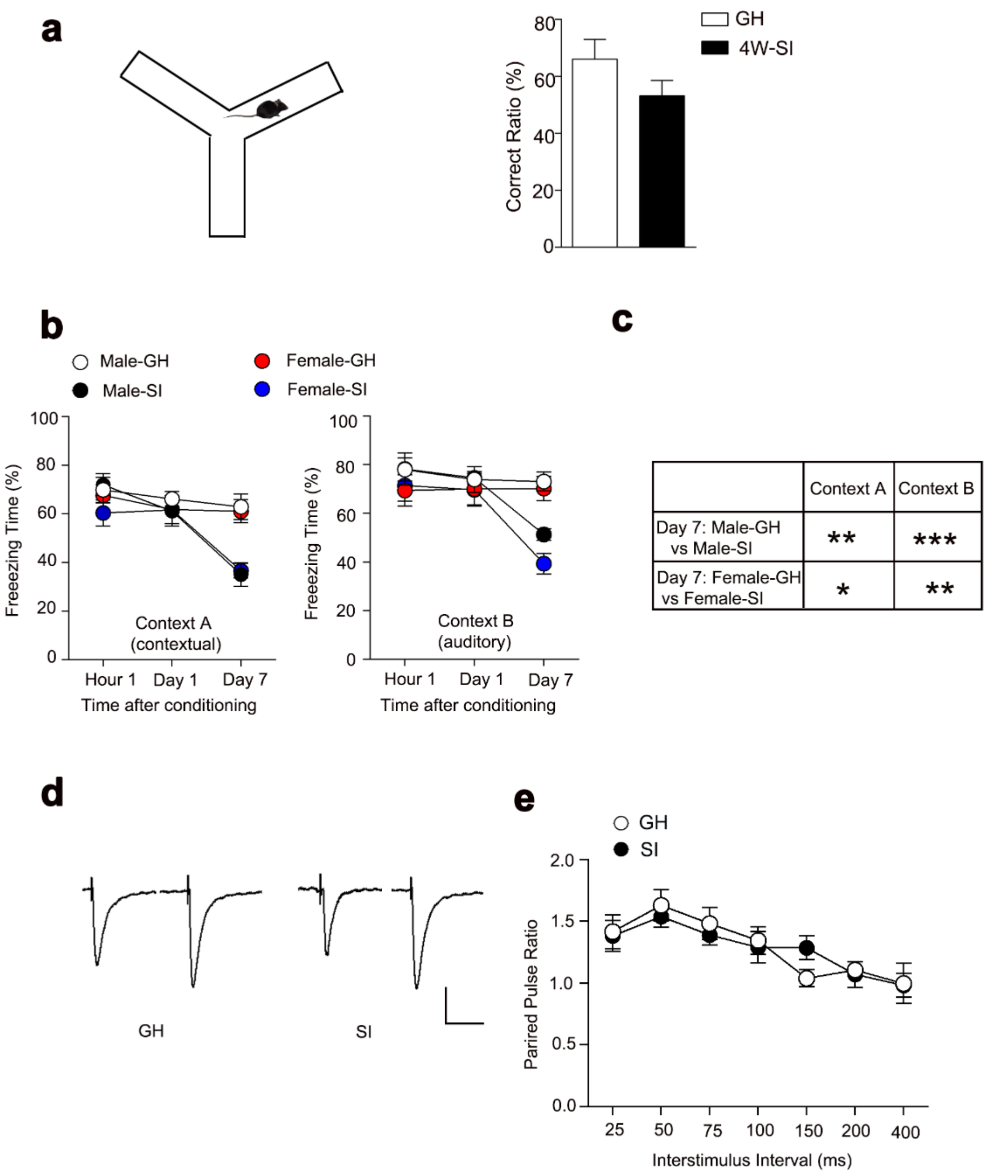

Supplementary Fig. 2 SI do not affect the fear extinction and presynaptic function. a No difference was seen in short-term memory or spatial memory between group housed mice and 4-week isolated mice in Y-maze. GH, *n* = 9 mice; SI, *n* = 7 mice, unpaired Student's *t* test, $t_{(14)} = 1.406$, $p > 0.05$. **b** No difference was seen between males and females on social isolation induced fear memory forgetting. **c** The list showed the statistical results. **d** Representative PPR trace at 100 ms interval . Calibration: 100 pA, 25 ms. **e** Quantification of PPR from GH and SI mice. GH, *n* =

16 neurons from 4 mice; SI, $n = 15$ neurons from 4 mice; two-way *ANOVA*; effect of interval time, $F_{(6, 203)} = 7.292$, $p < 0.0001$; effect of group, $F_{(1, 203)} = 0.0322$, $p > 0.05$; group × interval time, $F_{(6, 203)} = 0.5651$, $p > 0.05$, Tukey post hoc analysis was performed. All data are presented as mean ± SEM. *$p < 0.05$; **$p < 0.01$; ***$p < 0.01$.

38

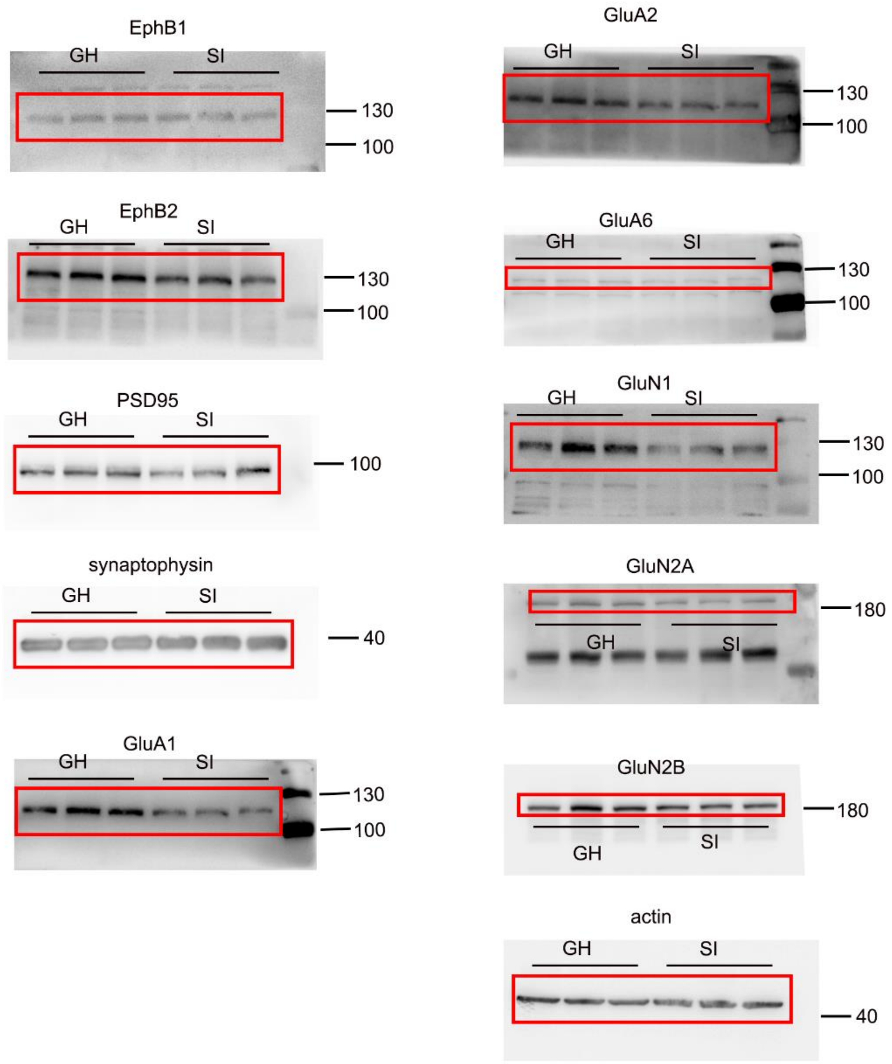

39

40 **Supplementary Fig. 3 Whole western blotting related to Fig. 2a.**

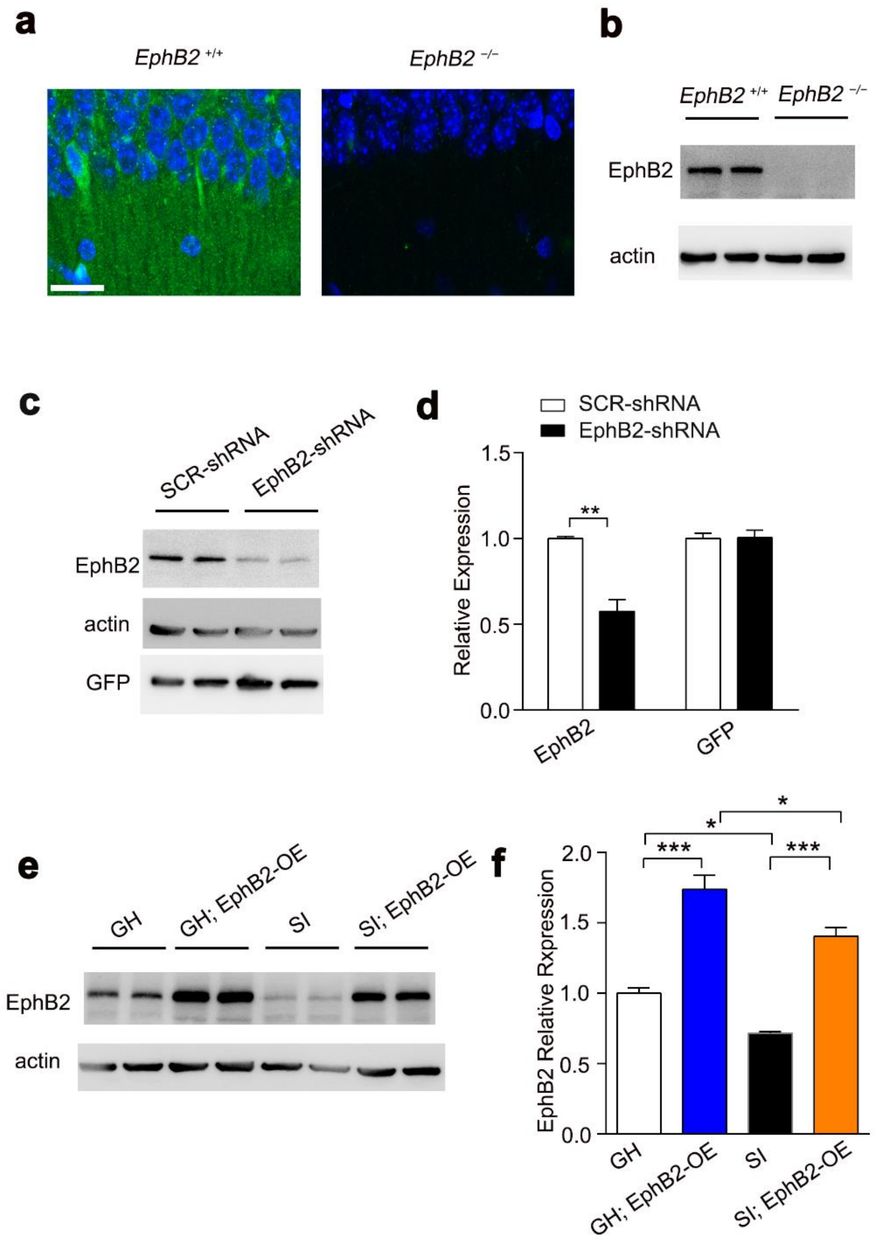

Supplementary Fig. 4 Verification of EphB2 antibody and virus. a Representative confocal image of EphB2 staining in *EphB2* $^{+/+}$ and *EphB2* $^{-/-}$ mice. Scale bar: 25 μm. **b** Western blotting showed the EphB2 and actin from *EphB2* $^{+/+}$ and *EphB2* $^{-/-}$ mice. **c** The expression of EphB2 in hippocampus CA1 region from SCR-shRNA and EphB2-shRNA mice were detected by western blotting. **d** The quantification of EphB2, GFP

proteins in hippocampus. $n = 3$ mice for each group, paired Student's t test, EphB2, $t_{(2)} = 5.991$, $p < 0.01$; GFP, $t_{(2)} = 0.0706$, $p > 0.05$. **e** The expression of EphB2 in hippocampus from GH, GH with AAV-EphB2 (EphB2-OE), SI, SI with AAV-EphB2 (EphB2-OE) mice was detected by western blotting. **f** The quantification of EphB2 proteins in hippocampus. $n = 3$ mice for each group, two-way *ANOVA*; effect of group, $F_{(1, 8)} = 24.79$, $p < 0.01$; effect of treatment, $F_{(1, 8)} = 132.1$, $p < 0.0001$; group × treatment, $F_{(1, 8)} = 0.1529$, $p > 0.05$; Tukey post hoc analysis was performed. All data are presented as mean ± SEM. *$p < 0.05$; **$p < 0.01$; ***$p < 0.01$.

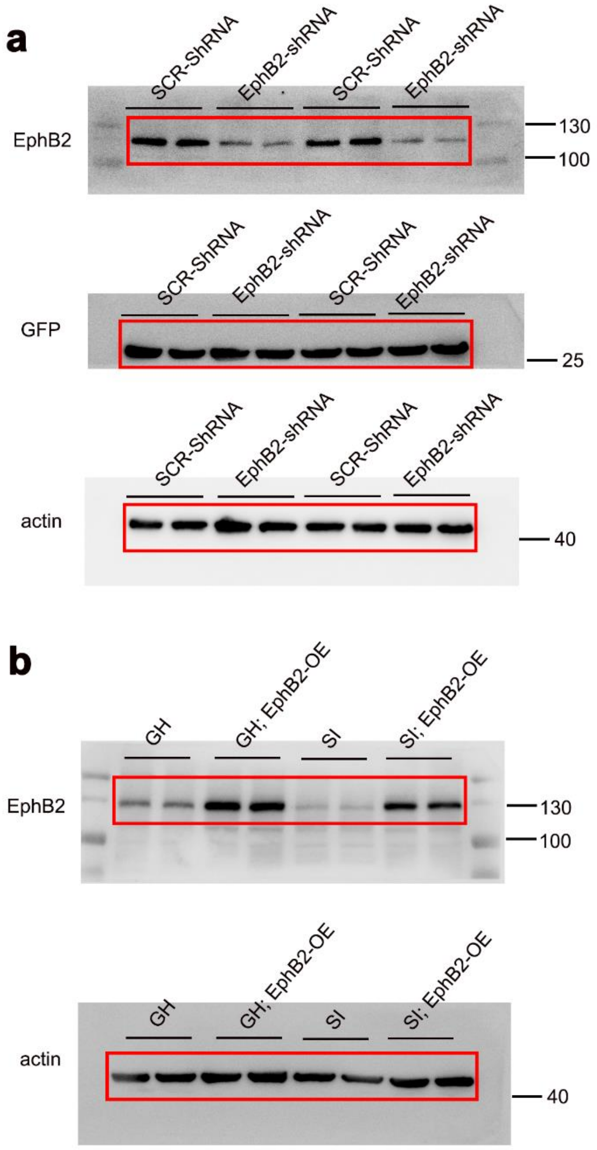

Supplementary Fig. 5 Whole western blotting related to Supplementary Fig. 3c (a) and 3e (b).

Supplementary Table 1. ***ANOVA*** **results related to Fig. 3b**

Context A

		F	*P*
Hour 1	group	$F_{(1,27)} = 0.04483$	$p > 0.05$
	treatment	$F_{(1,27)} = 0.7798$	$p > 0.05$
	interaction	$F_{(1,27)} = 0.1030$	$p > 0.05$
Day 1	group	$F_{(1,27)} = 0.7583$	$p > 0.05$
	treatment	$F_{(1,27)} = 0.2864$	$p > 0.05$
	interaction	$F_{(1,27)} = 0.6971$	$p > 0.05$
Day 7	group	$F_{(1,27)} = 11.92$	$p < 0.01$
	treatment	$F_{(1,27)} = 6.012$	$p < 0.05$
	interaction	$F_{(1,27)} = 1.054$	$p > 0.05$

Context B

		F	*P*
Hour 1	group	$F_{(1,27)} = 0.08326$	$p > 0.05$
	treatment	$F_{(1,27)} = 0.2988$	$p > 0.05$
	interaction	$F_{(1,27)} = 0.08326$	$p > 0.05$
Day 1	group	$F_{(1,27)} = 1.352$	$p > 0.05$
	treatment	$F_{(1,27)} = 0.4632$	$p > 0.05$
	interaction	$F_{(1,27)} = 0.02596$	$p > 0.05$
Day 7	group	$F_{(1,27)} = 9.527$	$p < 0.01$
	treatment	$F_{(1,27)} = 10.05$	$p < 0.01$
	interaction	$F_{(1,27)} = 2.749$	$p > 0.05$

Supplementary Table 2. ***ANOVA*** **results related to Fig. 3d**

		F	*P*
Spine density	group	$F_{(1,71)} = 1.56$	$p > 0.05$
	treatment	$F_{(1,71)} = 0.0005791$	$p > 0.05$
	interaction	$F_{(1,71)} = 0.7928$	$p > 0.05$
Mushroom	group	$F_{(1,71)} = 1.55$	$p > 0.05$
	treatment	$F_{(1,71)} = 10.62$	$p < 0.01$
	interaction	$F_{(1,71)} = 0.00514$	$p > 0.05$
Stubby	group	$F_{(1,71)} = 0.1221$	$p > 0.05$
	treatment	$F_{(1,71)} = 0.7599$	$p > 0.05$
	interaction	$F_{(1,71)} = 4.668$	$p < 0.05$
Thin	group	$F_{(1,71)} = 14.33$	$p < 0.001$
	treatment	$F_{(1,71)} = 12$	$p < 0.001$
	interaction	$F_{(1,71)} = 1.377$	$p > 0.05$

Supplementary Table 3. ***ANOVA*** **results related to Fig. 3f**

		F	*P*
Amplitude	group	$F_{(1,59)} = 14.25$	$p < 0.001$
	treatment	$F_{(1,59)} = 14.36$	$p < 0.001$
	interaction	$F_{(1,59)} = 3.168$	$p > 0.05$
Frequency	group	$F_{(1,59)} = 1.071$	$p > 0.05$
	treatment	$F_{(1,59)} = 0.09728$	$p > 0.05$
	treatment	$F_{(1,59)} = 1.28$	$p > 0.05$

Supplementary Table 4. ***ANOVA*** **results related to Fig. 3h**

		F	*P*
fEPSP slope	group	$F_{(3,319)} = 34.55$	$p < 0.001$
	treatment	$F_{(10,319)} = 0.01840$	$p > 0.05$
	interaction	$F_{(30,319)} = 0.02592$	$p > 0.05$

Supplementary Table 5. *ANOVA* results related to Fig. 4a

Context A

		F	*P*
Hour 1	group	$F_{(1,29)} = 0.034$	$p > 0.05$
	treatment	$F_{(1,29)} = 0.04654$	$p > 0.05$
	interaction	$F_{(1,29)} = 0.0001$	$p > 0.05$
Day 1	group	$F_{(1,29)} = 1.07$	$p > 0.05$
	treatment	$F_{(1,29)} = 1.249$	$p > 0.05$
	interaction	$F_{(1,29)} = 0.001656$	$p > 0.05$
Day 7	group	$F_{(1,29)} = 5.374$	$p < 0.05$
	treatment	$F_{(1,29)} = 6.836$	$p < 0.05$
	interaction	$F_{(1,29)} = 1.892$	$p > 0.05$

Context B

		F	*P*
Hour 1	group	$F_{(1,29)} = 0.08513$	$p > 0.05$
	treatment	$F_{(1,29)} = 0.01505$	$p > 0.05$
	interaction	$F_{(1,29)} = 0.3495$	$p > 0.05$
Day 1	group	$F_{(1,29)} = 0.3818$	$p > 0.05$
	treatment	$F_{(1,29)} = 0.1331$	$p > 0.05$
	interaction	$F_{(1,29)} = 0.06949$	$p > 0.05$
Day 7	group	$F_{(1,29)} = 10.73$	$p < 0.01$
	treatment	$F_{(1,29)} = 11.77$	$p < 0.01$
	interaction	$F_{(1,29)} = 5.916$	$p < 0.05$

Supplementary Table 6. ***ANOVA*** **results related to Fig. 4c**

		F	*P*
Spine density	group	$F_{(1,63)} = 5.565$	$p < 0.05$
	treatment	$F_{(1,63)} = 0.3292$	$p > 0.05$
	interaction	$F_{(1,63)} = 0.1663$	$p > 0.05$
Mushroom	group	$F_{(1,63)} = 0.3757$	$p > 0.05$
	treatment	$F_{(1,63)} = 0.05802$	$p > 0.05$
	interaction	$F_{(1,63)} = 4.972$	$p < 0.05$
Stubby	group	$F_{(1,63)} = 2.05$	$p > 0.05$
	treatment	$F_{(1,63)} = 1.447$	$p > 0.05$
	interaction	$F_{(1,63)} = 0.005215$	$p > 0.05$
Thin	group	$F_{(1,63)} = 5.578$	$p < 0.05$
	treatment	$F_{(1,63)} = 3.164$	$p > 0.05$
	interaction	$F_{(1,63)} = 8.956$	$p < 0.01$

Supplementary Table 7. *ANOVA* results related to Fig. 4e

		F	*P*
Amplitude	group	$F_{(1,66)} = 4.876$	$p < 0.05$
	treatment	$F_{(1,66)} = 8.257$	$p < 0.01$
	interaction	$F_{(1,66)} = 6.486$	$p < 0.05$
Frequency	group	$F_{(1,66)} = 0.4383$	$p > 0.05$
	treatment	$F_{(1,66)} = 0.6536$	$p > 0.05$
	treatment	$F_{(1,66)} = 2.266$	$p > 0.05$

Supplementary Table 8. *ANOVA* results related to Fig. 4g

		F	*P*
fEPSP slope	group	$F_{(3,330)} = 77.8$	$p < 0.0001$
	treatment	$F_{(10,330)} = 0.2552$	$p > 0.05$
	interaction	$F_{(30,330)} = 0.2882$	$p > 0.05$
